# Urine-based detection of *Leishmania donovani* antibodies using recombinant aurora kinase and otubain cysteine peptidase for noninvasive diagnosis of visceral leishmaniasis

**DOI:** 10.1128/jcm.00409-26

**Published:** 2026-09-25

**Authors:** Anirban Bhattacharyya, Mohd Kamran, Rudra Chhajer, Sarfaraz Ahmad Ejazi, Mehebubar Rahman, Rama Prosad Goswami, Major Madhukar, Vidya Nand Ravi Das, Pradeep Das, Krishna Pandey, Nahid Ali

**Affiliations:** 1 Infectious Diseases and Immunology Division, Council of Scientific and Industrial Research (CSIR)-Indian Institute of Chemical Biology 266658, Kolkata, West Bengal, India; 2 Department of Tropical Medicine, School of Tropical Medicine 76063, Kolkata, West Bengal, India; 3 Department of Molecular Biology, Rajendra Memorial Research Institute of Medical Sciences 83429, Patna, India; Mayo Clinic Minnesota, Rochester, Minnesota, USA

**Keywords:** aurora I-related kinase, otubain cysteine peptidase, visceral leishmaniasis, diagnosis, post-kala-azar dermal leishmaniasis, ELISA, dipstick

## Abstract

Visceral leishmaniasis (VL) is a fatal neglected tropical disease that requires early and accurate diagnosis to reduce mortality and interrupt transmission. Although parasitological confirmation using splenic aspirates is the gold standard, it is invasive and hazardous, while blood-based serological tests are often impractical in children and field settings. Consequently, noninvasive urine-based diagnostics represent an important unmet need. In the present study, we investigated the diagnostic utility of two novel *Leishmania donovani* antigens, otubain cysteine peptidase (LdOCP) and aurora I-related kinase (LdAIRK), using urine samples from VL-endemic regions of India. Both antigens play essential roles in parasite cell cycle progression and survival and are conserved among visceralizing *Leishmania* species. Urine specimens were collected from 135 individuals aged 10–60 years from Bihar and West Bengal between 2016 and 2024. Following preliminary immunoblot screening, antigen performance was validated using enzyme-linked immunosorbent assay and dipstick formats. Diagnostic accuracy was assessed using rK39 rapid diagnostic test results as the reference standard, and receiver operating characteristic analysis was performed. Both LdOCP and LdAIRK demonstrated 100% sensitivity (95% CI*:* 93.69–100%) and 100% specificity (95% CI: 86–100%). Mean optical density values were significantly higher in VL patients (0.491 ± 0.266 for LdOCP; 0.749 ± 0.200 for LdAIRK) than in controls (0.092 ± 0.046). Longitudinal analysis revealed a marked decline in antigen-specific antibody levels 6 months after treatment, indicating potential utility as test-of-cure markers. Concordant performance in dipstick assays supports feasibility for urine-based point-of-care diagnosis in resource-limited endemic settings and may aid treatment decisions where laboratory infrastructure is limited.

**IMPORTANCE:**

This study demonstrates that urine-based detection of *Leishmania donovani*-specific antibodies using LdOCP and LdAIRK provides a highly accurate, noninvasive approach for visceral leishmaniasis diagnosis and prognosis. The excellent sensitivity and specificity, together with a clear post-treatment antibody decline, support application in field and rural settings. Importantly, the proposed urine biomarker panel offers a scalable framework for future development of multiplex, instrument-free diagnostic platforms, facilitating simultaneous detection of multiple pathogens. These findings lay the groundwork for prospective clinical validation, integration into point-of-care workflows, and broader deployment for infectious disease surveillance in resource-limited endemic regions with significant translational public health impact potential.

## INTRODUCTION

Neglected tropical diseases collectively affect over 1 billion people worldwide, primarily impacting impoverished populations in tropical and subtropical regions (1). Visceral leishmaniasis (VL), or kala-azar, recognized as an NTD in 2015, remains one of the most severe forms of leishmaniasis and predominantly affects marginalized rural and peri-urban communities, often through cyclical outbreaks that strain fragile health systems (2). The disease ranges clinically from self-limiting cutaneous manifestations to fatal visceral involvement of organs such as the liver, spleen, and bone marrow (3). In endemic regions including India and Sudan, *Leishmania donovani* is transmitted via the anthroponotic cycle, with humans serving as the principal reservoir. In contrast, in Southern Europe, Northeastern Africa, and Central and South America, *Leishmania infantum* is primarily zoonotic, being transmitted from animal reservoirs to vectors and subsequently to humans (4). Transmission is sustained by active VL cases, individuals with asymptomatic carriers who silently perpetuate the infection cycle and post-kala-azar dermal leishmaniasis (PKDL) (5). Approximately 83% of global VL cases occur in countries like India, Brazil, Somalia, Kenya, Ethiopia, and Sudan (6). In India, VL remains endemic in Bihar, Jharkhand, West Bengal, and Uttar Pradesh, with 54 districts reporting active transmission as of March 2026 (7). Sustained control efforts have resulted in a dramatic decline in VL cases from 77,102 in 1992 to 599 in 2023, alongside a reduction in VL-related deaths from 1,419 to just 4 during the same period (7). Despite these gains, PKDL continues to threaten elimination efforts. Globally, PKDL is largely confined to six countries, with India accounting for approximately 62% of cases, followed by Sudan, Bangladesh, Kenya, South Sudan, Ethiopia, Nepal and Uganda (8). Within India, PKDL is reported from 54 districts across VL-endemic states, with 314 new cases documented in 2023 (9).

Timely diagnosis remains a major challenge for VL control, as early symptoms often mimic other endemic febrile illnesses such as malaria and dengue, leading to delayed treatment and increased morbidity and mortality (10). Conventional diagnostic methods based on microscopic detection of amastigotes in tissue aspirates are highly specific but invasive, technically demanding, and unsuitable for routine use in resource-limited settings (11). Molecular techniques enable detection of parasite DNA, particularly in cases with low parasite burden (10). Nonetheless, their sensitivity depends on the presence of parasite genetic material in clinical samples (2), and their widespread implementation remains limited in endemic regions due to high costs, technical complexity, and infrastructure requirements (12).

Rapid diagnostic tests (RDTs) based on the rK39 antigen have become central to VL diagnosis in the Indian subcontinent; however, antigenic heterogeneity among African *L. donovani* strains and cross-reactivity with co-endemic diseases limit their universal applicability (13). These challenges underscore the need for improved diagnostic biomarkers capable of accurately detecting active infection and confirming cure. Increasing attention has therefore focused on pathogen-derived protein antigens and anti-leishmanial antibodies that are produced during active infection and excreted in urine, an approach that has proven effective for VL diagnosis (14) (15). Urine-based diagnostics offer key advantages, including noninvasive sampling, operational simplicity, and the ability to reflect active disease processes, making them particularly suitable for large-scale screening and point-of-care testing in endemic settings (16). Urine is easy to collect and has been successfully evaluated as a diagnostic specimen for multiple infectious diseases, including schistosomiasis (17), leprosy (18), strongyloidiasis (19), and VL (20, 21). In the context of leishmaniasis, urine-based enzyme-linked immunosorbent assays (ELISAs) have been developed for VL diagnosis using *L. donovani* membrane antigens (LAg) (22, 23). However, the reliance on purified parasite antigens is associated with significant limitations, including batch-to-batch variability and high production costs, which hinder large-scale and standardized application (24).

Previously, we identified two *L. donovani* antigens: an otubain cysteine peptidase (LdOCP), a member of the deubiquitinase (DUB) family, and a putative aurora-like kinase (LdAIRK), a mitotic serine/threonine kinase (25, 26). LdOCP regulates protein turnover through deubiquitination, while LdAIRK displays dynamic mitotic localization, highlighting their essential roles in parasite cell cycle progression and survival (26, 27). To date, 20 DUBs across seven families, including otubains, have been identified in *Leishmania*, and both LdOCP and LdAIRK are conserved among visceralizing species and essential for both promastigote and amastigote stages (26, 27). Building on this, we evaluated the immunoreactivity of Ldocp and Ldairk using urine samples from VL and PKDL patients in India to assess their diagnostic performance and their potential to discriminate active infection from past exposure.

## MATERIALS AND METHODS

### Human participants

Urine specimens were obtained from participants with parasitologically confirmed VL (*n* = 57) and PKDL (*n* = 22). In addition, samples were obtained from endemic healthy controls (EHC, *n* = 11), defined as close relatives of VL patients residing in endemic areas, and from patients with other febrile illnesses (OD, *n* = 16) presenting with symptoms overlapping those of VL. The OD group included malaria (Ma, *n* = 6), tuberculosis (Tb, *n* = 2), viral fever (Vi, *n* = 6), and typhoid (Ty, *n* = 2). Twenty-four urine samples from non-endemic healthy controls (NEHCs) were obtained from participants at CSIR-Indian Institute of Chemical Biology (CSIR-IICB), West Bengal. Follow-up (FU) urine samples (*n* = 5) for longitudinal analysis were obtained from VL participants at three defined time points: prior to treatment initiation (day 0), at the end of therapy (day 30), and 6 months after the start of AmBisome treatment (day > 180). Clinical specimens were obtained from the Rajendra Memorial Research Institute of Medical Science (RMRIMS), Bihar, and the School of Tropical Medicine (STM), West Bengal. Diagnosis of VL in all cases was established through microscopic identification of parasites in splenic or bone marrow aspirate samples. PKDL cases were confirmed by PCR analysis of skin lesion specimens. In addition, both VL and PKDL cases were tested using the rK39 rapid diagnostic test (InBios International, Inc., Washington, USA). Diagnosis and case definitions were based on national guidelines issued by the Indian Council of Medical Research, Ministry of Health and Family Welfare, and the National Centre for Disease Control, Government of India (28).

All samples were collected as part of routine clinical evaluation and preserved in the Department of Infectious Diseases and Immunology, CSIR-IICB, between 2016 and 2025. For immunodiagnostic analyses, urine samples from VL and PKDL participants were considered positive controls, whereas samples from EHC, NEHC, and OD groups served as negative controls. Follow-up (FU) samples were analyzed to assess post-treatment antigen reactivity. Approximately 40 mL of midstream urine was collected in sterile 50 mL tubes, supplemented with 0.1% sodium azide as a preservative, and stored at 4°C until use.

### Cloning, expression, and purification of recombinant LdOCP and LdAIRK

The cloning of LdAIRK was performed as previously described (26). The procedure for cloning of LdOCP is detailed below. In brief, the LdOCP and LdAIRK genes derived from *L. donovani* strain AG83 (ATCC PRA-413) were amplified and subsequently cloned into the pET28a expression vector (Novagen, Madison, WI, USA) through NcoI and HindIII restriction sites (Promega, Madison, USA). The recombinant constructs were transformed into *Escherichia coli* Rosetta (DE3) cells and plated on Luria-Bertani (LB) agar (HiMedia Laboratories Pvt. Ltd., Mumbai, India) plates. The plates were maintained at 4°C for future protein expression experiments. For overexpression, freshly transformed cells (LdOCP) and a single colony from a plate stored at 4°C (LdAIRK) were cultured separately in 1 L of LB medium at 37°C. Protein expression of LdOCP and LdAIRK was induced using 0.3 mmol/L IPTG (Sigma-Aldrich, St. Louis, USA) once the culture reached an optical density (OD_600_) of 0.4–0.6. Prior to IPTG induction, 1 mL of culture was collected as an uninduced control. Following 4 h of induction, cells were harvested by centrifugation, and the pellets were resuspended in bacterial lysis buffer containing 25 mmol/L Tris-HCl, 300 mmol/L NaCl, 1 mg/mL lysozyme, and 1 mmol/L phenylmethylsulfonyl fluoride (PMSF) (Sigma-Aldrich), pH 8.0. Cells were lysed by sonication (Misonix, Farmingdale, NY, USA), and the resulting lysate was centrifuged at 12,000 rpm for 30 min to separate soluble and insoluble fractions. For Ldairk, which was predominantly expressed as inclusion bodies, the insoluble pellet was collected and solubilized in binding buffer containing 6 M urea and 10 mmol/L imidazole in 25 mmol/L Tris-buffered saline (TBS) (Sisco Research Laboratories Pvt. Ltd., Mumbai, India). The lysate was centrifuged at 11,000 rpm for 30 min, and the clarified supernatant was retained. In contrast, Ldocp was expressed as a soluble cytosolic protein and did not require urea solubilization; all subsequent purification steps were performed at 4°C. The supernatants were incubated with pre-equilibrated Ni-NTA agarose resin (Qiagen, Germany) to enable binding of the His-tagged recombinant proteins. The Ldairk-bound resin was washed twice with urea-containing buffer supplemented with 50 mmol/L imidazole and 0.1% Triton X-100, followed by two additional washes without Triton X-100. Recombinant Ldocp and Ldairk proteins were eluted using buffer containing 500 mmol/L imidazole (Sisco Research Laboratories Pvt. Ltd.). The urea-denatured Ldairk fractions were subjected to stepwise dialysis to gradually remove urea and facilitate proper protein refolding (24). Eluted proteins were concentrated with Amicon Ultra centrifugal filter units (Millipore Corporation, USA). The purity of the proteins was assessed by 10% SDS-PAGE and Coomassie Brilliant Blue staining, while protein concentration was quantified using the Lowry method (29).

### Amino acid sequence alignment

Template structures for homology modeling were identified through NCBI BLASTp searches against the Protein Data Bank, selecting candidates with the highest sequence identity to the target proteins. The protein amino acid sequences were used as query inputs to generate alignment profiles and identify suitable structural templates. To assess homology and interspecies conservation of LdOCP and LdAIRK, their amino acid sequences were compared across multiple *Leishmania* species, including *L. donovani, L. major, L. braziliensis, L. infantum,* and *L. mexicana*. Comparative analyses also included *Trypanosoma* species (*T. brucei* and *T. cruzi*) and *Homo sapiens*. Multiple sequence alignments (MSAs) were conducted to examine sequence conservation, and the resulting alignments were visualized using Jalview version 2.8 (University of Dundee, UK) (30).

### Immunoblot assay

Western blot analysis was performed to evaluate the immunoreactivity of recombinant Ldocp and Ldairk proteins. Proteins were separated by 10% SDS-PAGE and electrotransferred onto 0.45 µm nitrocellulose membranes (Bio-Rad, Germany) using a Bio-Rad Trans-Blot system (Bio-Rad Laboratories, CA, USA) at 1.5 A for 15 min. Transfer efficiency was confirmed by Ponceau S staining (Sigma-Aldrich). Membranes were rinsed with TBS to remove excess stain and cut into individual strips corresponding to each lane. Strips were blocked in 5% bovine serum albumin (BSA) (GOLDBIO, St. Louis, USA) prepared in Tris-buffered saline (TBS) for 1 h at room temperature. Following washes with TBST (TBS containing 0.05% Tween-20), the strips were incubated overnight at 4°C with gentle agitation in primary antibodies obtained from urine samples diluted 1:10 (20). After three washes with TBST, strips were incubated with horseradish peroxidase (HRP)-conjugated goat anti-human IgG secondary antibody (Southern Biotech, Birmingham, USA) diluted 1:3,000 for 1 h at room temperature with continuous shaking. Strips were then washed five times with TBST at 5-min intervals, followed by a final wash with TBS. Chemiluminescent HRP substrate (Sigma-Aldrich) was applied to the strips, and signals were detected using a Gel Doc imaging system (Bio-Rad Laboratories, Inc.). Images were acquired within 20 s of substrate application, using Image Lab software version 5.4.2 (Bio-Rad) (31).

### Enzyme-linked immunosorbent assay (ELISA)

To detect IgG antibodies specific to recombinant Ldocp and Ldairk, an indirect ELISA was carried out. Each antigen solution (1 mg/mL) was coated onto 96-well ELISA plates (Nunc) at a volume of 100 µL per well in 10 mmol/L phosphate buffer (PB). After overnight incubation at 4°C, plates were washed twice with PBST (phosphate-buffered saline containing 0.05% Tween-20) and blocked with 200 µL of 1% BSA in PBS per well for 1 h at 37°C. Subsequently, wells were washed three times with PBST, followed by the addition of urine samples diluted 1:10 (21) as the primary antibody source (100 µL per well in PBST supplemented with 2% BSA) and incubated for 2 h at 37°C. Plates were subsequently incubated with HRP-conjugated goat anti-human IgG secondary antibody diluted 1:3,000 in PBST for 2 h at 37°C. Following five PBST washes, antibody binding was visualized by adding 100 µL of 3,3′,5,5′-tetramethylbenzidine (TMB) substrate (Sigma-Aldrich) per well. The color reaction was developed for about 2 min and stopped with 50 µL of 2 N H_2_SO_4_ to each well. Optical density was recorded at 450 nm using a microplate spectrophotometer (Thermo Fisher Scientific, USA).

### Dipstick preparation and assay

For the development of dipstick devices for field-based diagnostics, nitrocellulose membranes (2.4 × 8 cm; 0.45 µm pore size; GE Healthcare Life Sciences) were pre-soaked in 25 mmol/L Tris-HCl buffer (pH 7.6). After partial drying, recombinant Ldocp and Ldairk antigens were dispensed onto the membrane as test lines at 1 µg per line with a dispensing rate of 5 µL/cm. In parallel, unlabeled rabbit anti-human IgG (Southern Biotech) diluted 1:10 was dispensed onto the control line using a Flowline F100 dispenser (Precore Solutions, Kochi, India) to act as an internal positive control (32). Then, after a 30-min incubation at room temperature, membranes were blocked overnight at 4°C in blocking buffer containing 2% BSA, 0.1% Tween-20, and 0.01% NaN_3_ in 100 mmol/L TBS. Membranes were then washed with TBST, air-dried, attached to plastic backing sheets, and sectioned into 0.4-cm-wide dipsticks. The prepared dipsticks were stored under desiccated conditions at room temperature and maintained stability for up to 1 year. For analysis, dipsticks were placed in 1.5 mL vials containing urine samples diluted 1:10 in TBS (20). Following sample incubation, strips were treated with HRP-conjugated goat anti-human IgG secondary antibody diluted 1:2,000. Dipsticks were then washed twice with TBST and once with TBS to remove excess reagents. Color development was achieved by immersing the strips in substrate solution containing 0.05% 3,3′-diaminobenzidine tetrahydrochloride (DAB; Sigma) and 0.05% H_2_O_2_ prepared in 100 mmol/L TBS. The reaction was terminated by rinsing the dipsticks with distilled water (33).

### Diagnostic procedure using rK39 RDTs

The rK39 RDT is a qualitative immunochromatographic assay that detects antibodies directed against the *Leishmania* complex (*L. donovani and L. infantum*) in whole blood, serum, or urine samples. A total of 61 Kalazar Detect rK39 Rapid Test kits (InBios International, Inc.) were used in this study and performed strictly according to the manufacturer’s instructions. The appearance of a red line in the test region, irrespective of intensity, was interpreted as a positive result, as specified by the manufacturer. Each assay was recorded as either positive or negative. All tests were performed by a single operator who was not blinded to the reference diagnostic results.

### Data analysis

Receiver operating characteristic (ROC) curve analysis and statistical evaluations were carried out using GraphPad Prism version 8.0 (Boston, USA). ROC curves were generated with 95% confidence intervals to compare VL and healthy control (HC) groups and to determine optimal cutoff values that maximized both sensitivity and specificity. Sensitivity was defined as the proportion of VL samples correctly classified as positive, whereas specificity represented the proportion of non-VL samples (NEHC, EHC, and OD) correctly classified as negative. Diagnostic performance was assessed by calculating the area under the ROC curve (AUC), where higher AUC values indicate superior discriminative ability, and an AUC of 1.0 reflects perfect diagnostic accuracy. Statistical differences between groups were analyzed using the Mann–Whitney *U*-test, with a *P* value < 0.05 considered statistically significant (34).

## RESULTS

### Sequence analysis, cloning, and purification of *L. donovani* LdOCP and LdAIRK

**Fig 1:**
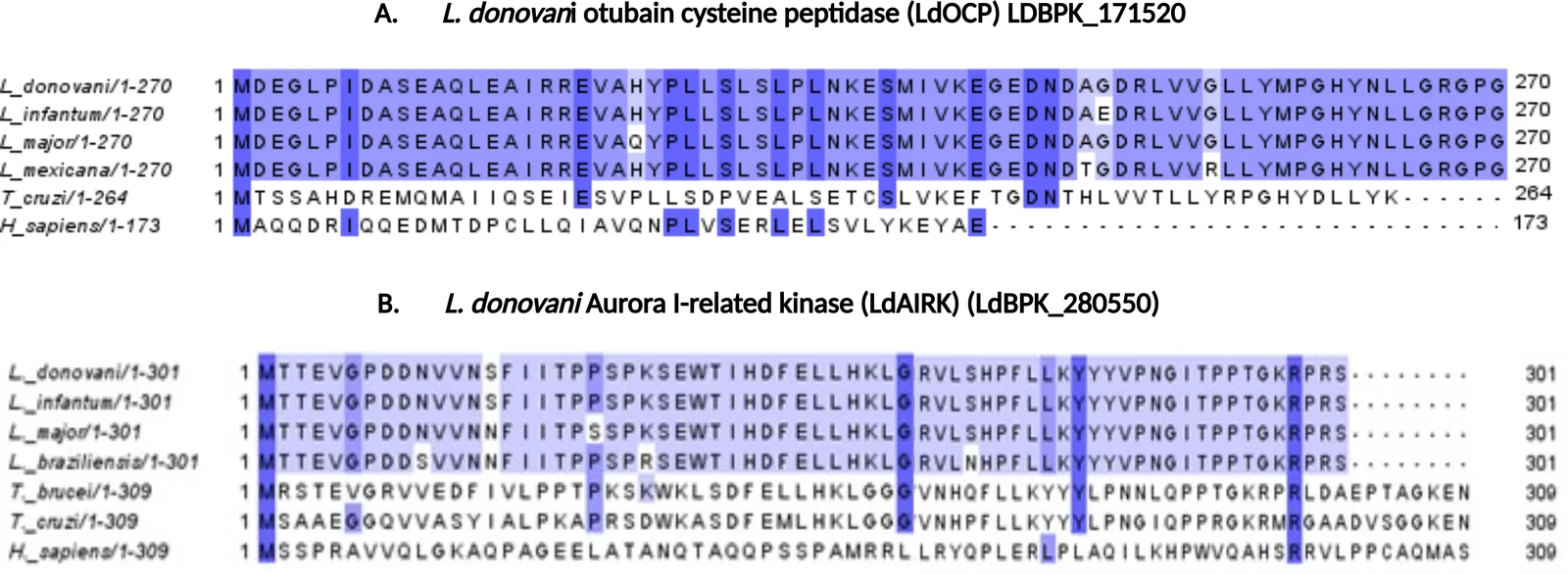
Sequence comparison of (LdOCP) LDBPK_171520 and LdAIRK (LdBPK_280550). Multiple sequence alignment of amino acid sequences was performed using Jalview 2.8 to compare LdOCP and LdAIRK homologs from *L. donovani*, *L. infantum*, *L. major*, *L. braziliensis*, *L. mexicana*, *T. brucei*, *T. cruzi*, and *H. sapiens*. Part of conserved residues was shown and are highlighted with a purple background, with the intensity of the color reflecting the degree of conservation. The numbers on the left and right indicate the positions of the amino acids.

**Fig 2:**
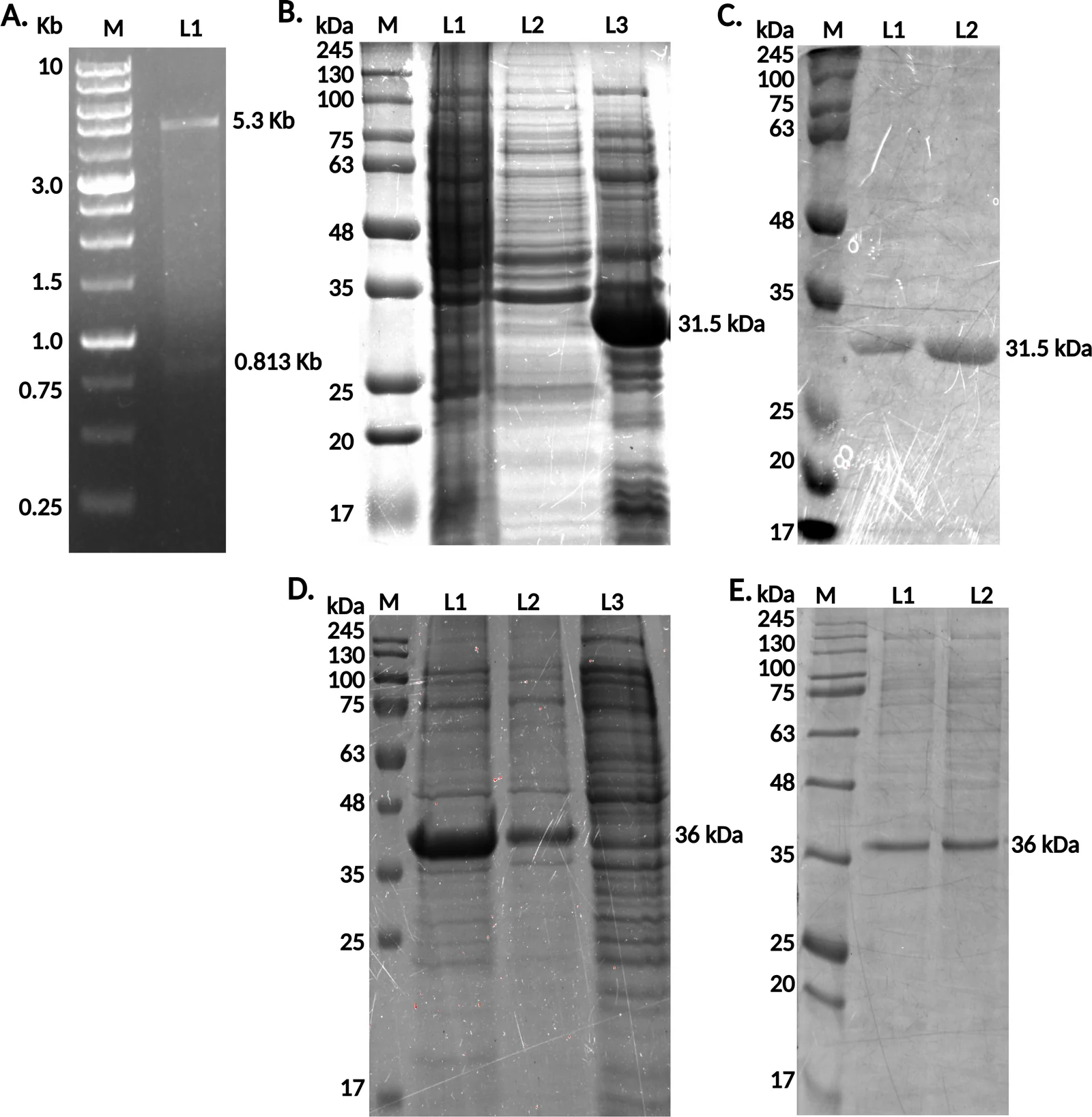
Cloning of LdOCP, expression of purified recombinant LdOCP and LdAIRK. (**A**) LdOCP was PCR amplified and cloned into pET28a. Lane M shows base pair markers, with lane L1 showing the 5.3 kb pET28a and 0.813 kb LdOCP bands, respectively. (**B**) and (**D**) LdOCP and LdAIRK, respectively, expression was induced with 0.3 mM IPTG. In panel **B**, lanes L1 and L2 show uninduced lysates, whereas lane L3 shows the induced 31.5 kDa recombinant LdOCP protein. In panel **D**, lanes L1 and L2 show the induced 36 kDa recombinant LdAIRK protein, whereas lane L3 shows the pre-induction lysate. (**C**) and (**E**) Purification of Ldocp and Ldairk respectively. Lanes L1 and L2 display purified recombinant proteins. Lane M shows molecular markers.

A complete open reading frame (ORF) of *L. donovani* LdOCP (LDBPK_171520) and LdAIRK (LDBPK_280550), comprising 813 and 906 bp, respectively, was retrieved from the TriTrypDB database. These genes encode proteins of 270 and 301 amino acids, with predicted molecular weights of approximately 31.5 kDa (LdOCP) and 36 kDa (LdAIRK) (Table 1). Multiple sequence alignment (MSA) analysis highlighted the homology and interspecies conservation of LdOCP and LdAIRK among various kinetoplastid species and *H. sapiens*. Conserved residues are shown in purple, with color intensity corresponding to the degree of conservation (Fig. 1). Both proteins were highly conserved among *Leishmania* species but exhibited lower similarity with *Trypanosoma* species and humans. Notably, LdOCP displayed greater conservation (higher color intensity) compared to LdAIRK. Amino acid sequences of the BPK strain of *L. donovani* were obtained from the NCBI database, followed by BLAST analysis against genomic databases of visceralizing *Leishmania* species, *T. cruzi*, and *H. sapiens* (Table 2). LdOCP and LdAIRK showed high sequence identity (97–98%) with *L. infantum* and *L. donovani*, whereas significantly lower identity was observed with *T. cruzi* (36–39%) and *H. sapiens* (32–43%). For cloning, PCR amplification of the LdOCP gene was performed using gene-specific primers on genomic DNA from *L. donovani* strain AG83. A distinct 0.813 kb amplicon corresponding to the expected gene size was obtained, confirming successful amplification (Fig. 2A). The amplified product was subsequently cloned and expressed in *E. coli* Rosetta (DE3) cells following IPTG induction (Fig. 2B). Maximal expression of Ldocp was observed in lane 3 compared to the uninduced controls (lanes 1 and 2). SDS-PAGE analysis of the purified recombinant protein revealed a band at the expected molecular weight of approximately 31.5 kDa in the elution fractions (Fig. 2C). The LdAIRK clone, generated in a previous study (24), was similarly expressed and induced in *E. coli* (Fig. 2D). Induced samples showed expression (lanes 1 and 2) compared to the uninduced control in lane 3. Purified recombinant Ldairk protein migrated at the expected molecular weight of approximately 36 kDa in the elution fractions (Fig. 2E).

**TABLE 1:** GenBank accession numbers of the identified targets’ nucleotide and protein sequences

Gene and accession number	Nucleotide sequences	Protein sequences	Base pair (kb)	Protein length (aa)	Molecular weight
*L. donovani* otubain cysteine peptidase (LdOCP) LDBPK_171520	XM_003859959	XP_003860007.1	813	270	31.5 kDa
*L. donovani* aurora I-related kinase (LdAIRK) (LdBPK_280550)	XM_003862106	XP_003862154	906	301	36 kDa

**TABLE 2:** In silico comparison of Ldocp and Ldairk genes with *Trypanosoma cruzi*, *Homo sapiens*, and *Leishmania* strains linked to visceral leishmaniasis

GENE and Acc. number	Species	Accession	Percent identity (%)
*L. donovani* otubain cysteine peptidase (LdOCP) LDBPK_171520	*L. donovani*	XP_003860007.1	97.78
	*L. infantum*	XP_001464809.1	97.78
	*T. cruzi*	XP_816378.1	39.15
	*H. sapiens*	EAW74187.1	31.86
*L. donovani* Aurora I-related kinase (LdAIRK) (LdBPK_280550)	*L. donovani*	XP_003862154.1	97.01
	*L. infantum*	XP_001470088.1	97.01
	*T. cruzi*	EKG02707.1	36.69
	*H. sapiens*	NP_001015878.1	42.81

### Immunoblot assay-based Ldocp and Ldairk validation with urine samples

**Fig 3:**
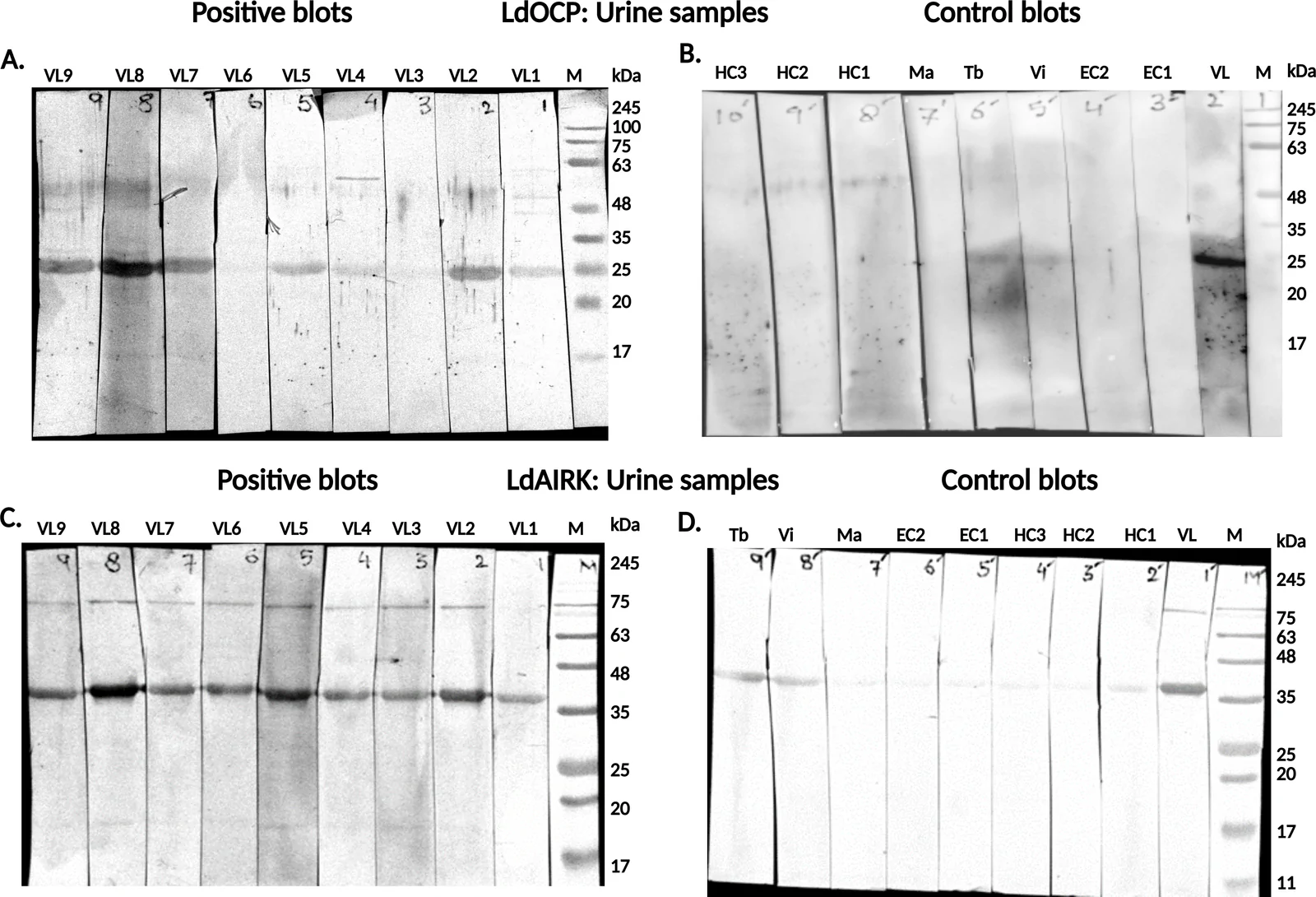
Immunoblot assay of recombinant Ldocp and Ldairk antigens was performed using urine samples from India. Panels (**A**) and (**C**) show results for nine VL-positive samples (VL1–VL9) with protein markers in lane M. Panels (**B**) and (**D**) display control samples: non-endemic (HC1–HC3), endemic healthy controls (EC1 and EC2), other diseases (TB, Ma, and Vi), and VL-positive controls (VL), with lane M containing the protein marker.

The immunoreactivity of recombinant Ldocp and Ldairk antigens was assessed by Western blot analysis using four independent immunoblots (two per antigen) probed with urine samples from VL-positive patients and healthy controls. Both antigens demonstrated strong reactivity with urine from nine VL patients, consistently yielding bands at approximately 31.5 kDa (Ldocp) and 36 kDa (Ldairk) in positive blots (Fig. 3A through C). In contrast, no significant reactivity was observed when membranes were probed with urine from two endemic and three non-endemic healthy controls. Similarly, no binding was detected with malaria samples. However, faint cross-reactivity was observed with viral fever and tuberculosis samples. As expected, strong positive reactivity was consistently observed with one VL patient sample in control blots (Fig. 3B through D).

### Performance of Ldocp- and Ldairk-based ELISAs using urine samples

**Fig 4:**
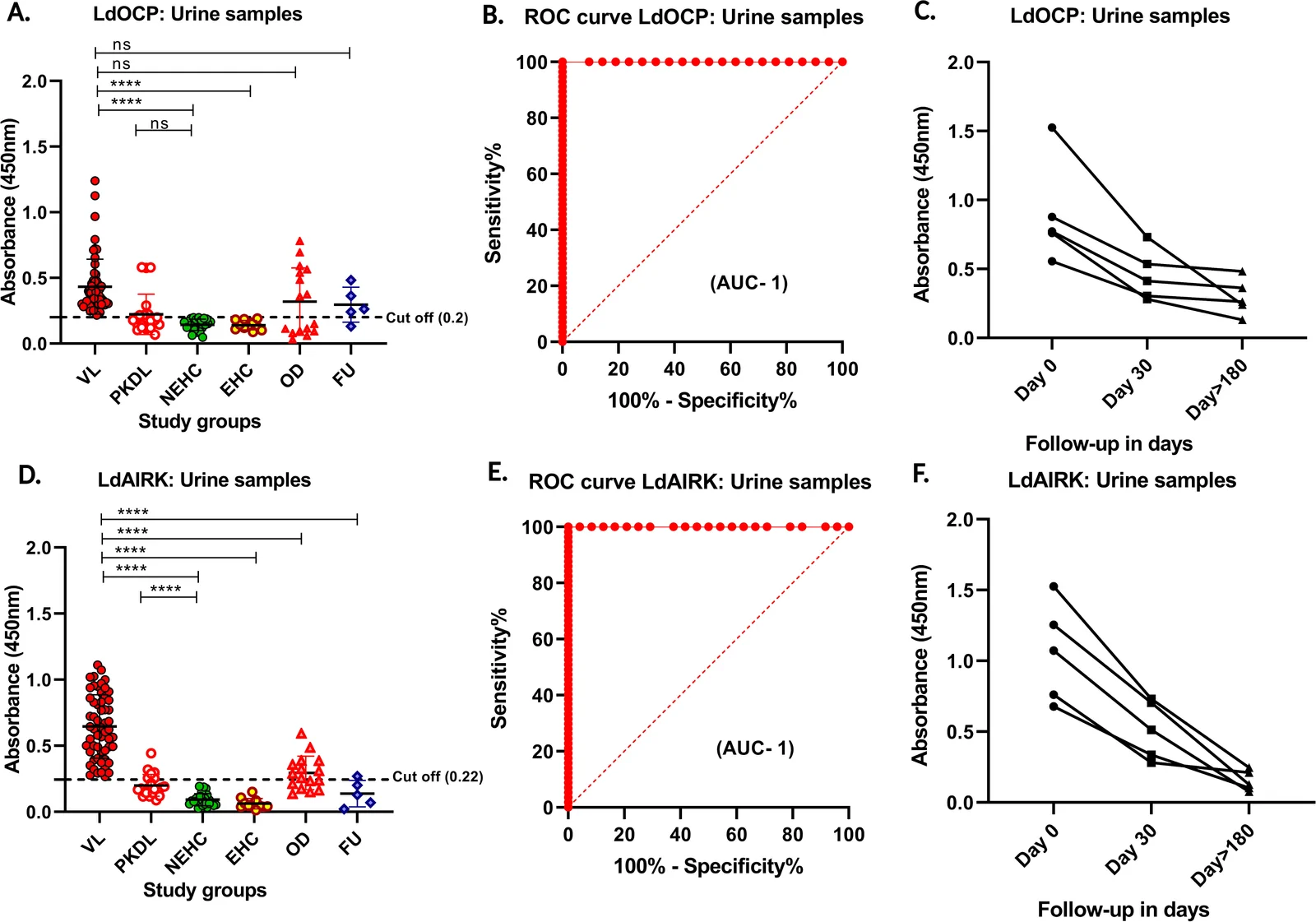
Indian urine-based ELISA was conducted using recombinant Ldocp and Ldairk antigens. Urine samples were collected from VL patients (*n* = 57), PKDL cases (*n* = 22), NEHC participants (*n* = 24), EHC participants (*n* = 12), OD cases (*n* = 19), and FU cases (*n* = 5). Dotted lines in panels (**A**) and (**D**) indicate cutoff values for Ldocp (0.2) and Ldairk (0.22), respectively, determined using ROC curves for optimal sensitivity and specificity (****P < 0.0001). Panels (**B**) and (**E**) show ROC curves for detecting antigen-specific antibodies in urine samples using the Ldocp and Ldairk antigens. Panels (**C**) and (**F**) compare the levels of antibodies in VL urine samples from India before treatment started and post-treatment. Absorbance values were measured at three time points: before treatment (day 0), 1 month after treatment (day 30), and at least 6 months’ post-treatment (day 180) for Ldocp and Ldairk antigens. Paired *t*-tests were conducted to compare antibody levels at different time points.

Following confirmation of immunogenicity by immunoblotting, the diagnostic performance of Ldocp and Ldairk was further evaluated in an expanded and diverse cohort using indirect ELISA. The cutoff values for both Ldocp-based and Ldairk-based ELISAs were established at 0.2 based on the ROC curve analysis and the distribution of absorbance values among healthy control samples. The AUC for both assays was 1.0. The mean optical density (OD) values for VL patients were 0.491 ± 0.266 (Ldocp) and 0.749 ± 0.200 (Ldairk), which were significantly higher than those observed in controls (0.084 ± 0.082 for Ldocp and 0.092 ± 0.046 for Ldairk). Sensitivity for VL detection was 100% for both antigens (95% CI: 93.69–100%), indicating correct identification of all VL cases. Specificity was also 100% (95% CI: 86–100%) for both antigens within the Indian control groups (Fig. 4A through D and Table 3). In contrast, reduced sensitivity was observed for PKDL detection, with values of 31.82% (95% CI: 16.36–52.68%) for Ldocp and 36.36% (95% CI: 19.73–57.05%) for Ldairk. Among EHC, both antigens demonstrated 100% specificity. However, in samples from patients with diseases clinically similar to VL, specificity decreased to 50% (95% CI: 28–72%) for Ldocp and 31.25% (95% CI: 14.16–55.60%) for Ldairk. Statistical analysis using the Mann–Whitney *U*-test and ROC curve assessment further supported the strong discriminatory performance of both Ldocp-based and Ldairk-based ELISAs for distinguishing VL urine samples from healthy controls (Fig. 4B through E), highlighting their diagnostic potential for VL detection.

**TABLE 3:** Assessment of recombinant LdOCP-based and LdAIRK-based ELISAs and dipsticks for VL detection in urine samples from India

		Urine samples, No./Total, 95% confidence interval								
Test sample-type	Antigen used	VL	PKDL	NEHC	EHC	OD	Total control	VL follow-up	AUC value	Cutoff value
		(Sensitivity, %)		(Specificity, %)				(Reactivity, %)		
ELISA-URINE	LdOCP	100 (57/57) 93.69– 100	31.82 (06/22) 16.36–52.68	100 (24/24) 86.2– 100	100 (11/11) 74.12– 100	50 (8/16) 28–72	84.31 (43/51) 71.99– 91.83	80 (4/5)^*a*^	1	0.20
	LdAIRK	100 (57/57) 93.69– 100	36.36 (08/22) 19.73–57.05	100 (24/24) 86.68– 100	100 (11/11) 74.12– 100	31.25 (05/16) 14.16–55.60	78.43 (40/51) 65.37– 87.51	20 (1/5)^*a*^	1	0.22
DIPSTICK-URINE	LdOCP	100 (30/30)	50 (6/12)	100 (20/20)	100 (10/10)	90 (9/10)	97.5 (39/40)	40 (2/5)		
	LdAIRK	100 (30/30)	83.33 (10/12)	100 (20/20)	100 (10/10)	100 (10/10)	100 (40/40)	20 (1/5)		
RDT-URINE rK39		100 (20/20)	83.33 (05/06)	100 (10/10)	70 (07/10)	80 (08/10)	83.33 (25/30)	80 (4/5)		

^*a*^ Signifies that there is no data.

### Utility of Ldocp and Ldairk antigen detection ELISA test in monitoring VL treatment

To assess treatment response, antigen-specific IgG levels against Ldocp and Ldairk were measured in urine samples from Indian FU cases. ELISA analysis using the Mann-Whitney *U*-test demonstrated a significant reduction in antibody reactivity at the follow-up stage. Antigen positivity in post-treatment samples was observed in 80% of samples for Ldocp and 20% for Ldairk (Fig. 4A through D), compared with the markedly higher antibody levels detected prior to treatment. Although a few FU samples marginally exceeded the established cutoff value of 0.2, overall reactivity remained low, indicating a substantial decline in antigen-specific antibody levels following therapy (Table 3). For longitudinal evaluation, paired urine samples from five *Leishmania-*infected patients were analyzed at three time points: before treatment, 1 month after treatment, and ≥6 months post-treatment (Fig. 4C through F). Antibody responses to both antigens declined sharply by 30 days after treatment initiation and continued to decrease, reaching minimal levels by 180 days post-treatment.

### Performance of Ldocp- and Ldairk-based dipstick assay with urine samples

**Fig 5:**
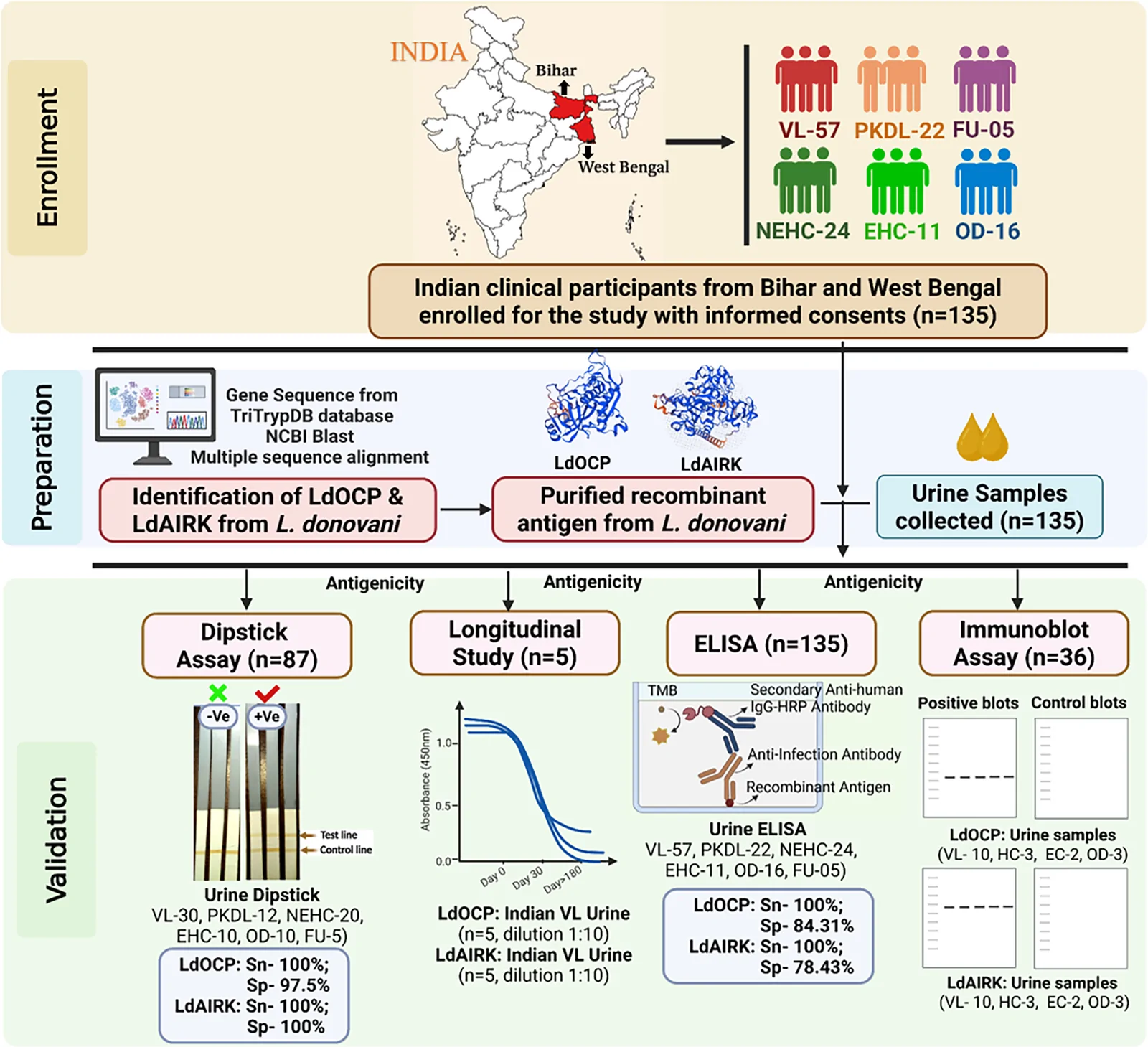
A proposed diagnostic flowchart for Indian participants, including visceral leishmaniasis (VL), post-kala-azar dermal leishmaniasis (PKDL), endemic healthy controls (EHCs) from endemic regions, and non-endemic healthy controls (NEHCs), utilizing urine samples collected from West Bengal and Bihar, India, and evaluating their antigenicity with recombinant Ldocp and Ldairk antigens through immunoblotting, ELISA, dipstick assays, and longitudinal studies. Representative dipstick assay images based on recombinant Ldocp and Ldairk antigens are shown from experiments performed in the present study, whereas the schematic illustrations of longitudinal studies, ELISA, and immunoblotting are illustrative in nature and intended solely to depict the overall study design.

Following confirmation of immunogenicity by immunoblotting and ELISA, the diagnostic applicability of Ldocp and Ldairk was further assessed using a field-adaptable dipstick format. Urine samples from VL patients in India produced intense bands at both the test and control lines on the nitrocellulose strips, yielding a sensitivity of 100% for both antigens. In contrast, urine samples from Indian negative controls (NEHC, EHC, and OD) showed bands exclusively at the control line, with no detectable reactivity at the test line. This resulted in a specificity of 100% for both Ldocp and Ldairk in the Indian control cohort. FU samples demonstrated reduced reactivity. With the Ldocp-based dipstick, 40% of treated cases remained positive, whereas only 20% showed reactivity with the Ldairk dipstick. A comparative summary and diagnostic flowchart of the analytical performance of the Ldocp-based and Ldairk-based dipstick assays with ELISA is presented in Fig. 5 and Table 3.

### Evaluation of Ldocp-based and Ldairk-based dipstick assay against reference rK39 ICTs in urine samples

Following the successful development of the Ldocp-based and Ldairk-based dipstick assays, their diagnostic performance was compared with that of a commercially available rK39 ICT (Kalazar Detect, InBios International). The comparison included urine samples from 20 confirmed VL cases, 6 PKDL cases, 30 control individuals (10 each from NEHC, EHC, and OD groups), and 5 FU cases from India. Both Ldocp-based and Ldairk-based dipstick assays demonstrated 100% sensitivity for VL cases and 100% specificity for NEHC samples, comparable to the rK39 ICT. However, in PKDL cases, the rK39 test showed slightly reduced sensitivity (83.33%), which was comparable to that of the Ldairk dipstick. Moreover, in the EHC and OD groups, both Ldocp-based and Ldairk-based dipsticks achieved high specificity ranging from 90% to 100%, whereas the rK39 ICT demonstrated lower specificity of 70% for EHC and 80% for OD. Overall, the total specificity of the rK39 ICT was 83.33%, as 5 out of 30 control samples showed positive reactivity with both test and control lines. In contrast, the total specificity of the Ldocp-based and Ldairk-based dipstick assays ranged from 98% to 100%. Interestingly, analysis of post-treatment FU samples showed that the rK39 ICT retained 80% reactivity, whereas reactivity with the Ldairk dipstick was reduced to 20% (Table 3).

## DISCUSSION

The identification of reliable clinical biomarkers for VL remains a critical unmet need in the management of kala-azar (35). Early diagnosis and timely therapeutic intervention are essential for improving patient outcomes, particularly in cases complicated by systemic and renal involvement (36). Although splenic aspiration remains the diagnostic gold standard, its invasive nature limits its utility for routine surveillance and follow-up. Consequently, the discovery and validation of noninvasive, sensitive, and specific biomarkers capable of predicting active infection and monitoring treatment response are of paramount importance (37). In the present study, we validated two *L. donovani* proteins otubain cysteine peptidase (LdOCP; XP_003860007) and aurora I-related kinase (LdAIRK; XP_003862154), as diagnostic biomarkers for VL. Both are expressed in promastigote and amastigote stages, supporting their relevance during mammalian infection. In *Leishmania*, ubiquitin-like modifiers (Atg8 and Atg12) and deubiquitinases (DUBs) regulate autophagy and are essential for promastigote-to-amastigote differentiation and intracellular survival (27, 38). Moreover, inhibition of Aurora kinase B disrupts G2/M progression and spindle organization in *L. donovani*, inducing mitochondrial dysfunction and mitotic catastrophe-like death (39). Together, these findings highlight the biological and clinical significance of DUBs and aurora kinases as diagnostic and therapeutic targets in VL. Given the conserved biological roles of LdOCP and LdAIRK in parasite survival and cell-cycle regulation, we further investigated their sequence conservation across visceralizing *Leishmania* species prevalent in different endemic regions. BLAST analysis revealed that both proteins share high sequence identity (approximately 97%) with *L. infantum*, the primary etiological agent of VL in the Mediterranean basin, Latin America, and parts of Africa, while showing minimal homology with *T. cruzi* and *H. sapiens*. Notably, LdOCP exhibited slightly higher conservation (97.78%) than LdAIRK (97%), as reflected by the greater intensity of conserved residues across *Leishmania* species in the sequence alignment. Given their high level of conservation among *Leishmania* species, these findings underscore the potential applicability of these antigens for developing diagnostic assays not only for *L. donovani* in India and East Africa but also for *L. infantum* associated VL in other endemic regions worldwide. Following cloning, expression, and purification of the recombinant proteins, we evaluated their antigenicity using urine samples from VL and PKDL patients from India through immunoblotting, ELISA, and dipstick assays. Recombinant LdOCP and LdAIRK demonstrated strong and specific recognition of anti*-L*. *donovani* IgG antibodies in urine, supporting their utility as urine-based diagnostic targets.

Recombinant kinesin-derived antigen rK39 has long served as a cornerstone of VL serodiagnosis and remains a reference test in the Indian subcontinent (40). However, substantial regional variability in rK39 performance has been reported, with reduced sensitivity in East Africa and Brazil, compared to South Asia (40). Moreover, these tests cannot differentiate active from past infection, as parasite-specific antibodies may persist for up to 2 years after treatment (40). Furthermore, urine-based rK39 assays demonstrate high sensitivity but suboptimal specificity (approximately 68%) in Indian settings, likely due to cross-reactivity with other infectious diseases such as malaria and enteric fever (41). Similarly, the kala-azar latex agglutination test (KAtex), urine antigen test, while highly specific, exhibits inconsistent sensitivity (47.7–85.7%) and limited reproducibility due to the use of undefined antigens (42). These limitations highlight the need for standardized recombinant antigens that provide consistent performance across endemic settings.

Our evaluation of recombinant Ldocp and Ldairk began with membrane-based immunoblot assays, which are critical for assessing antigen–antibody interactions. Both antigens demonstrated strong reactivity with urine samples from VL patients in India, while only mild reactivity was observed with control samples, including those from patients with other infectious diseases. Subsequent ELISA-based evaluation revealed excellent diagnostic performance for both antigens, with 100% (95% CI: 93.69–100%) sensitivity for detecting anti-*Leishmania* antibodies in urine samples from VL patients. Although the specificity of both antigens against NEHC samples was 100% (95% CI: 86.68–100%), the overall specificity was lower for Ldairk (78.43%; 95% CI: 65.37–87.51%) compared with Ldocp (84.31%; 95% CI: 71.99–91.83%). This reduced specificity of Ldairk was attributed to cross-reactive binding with a few samples from other diseases. Notably, Ldairk exhibited superior binding capability in urine samples from PKDL patients compared to Ldocp. The use of urine as a diagnostic specimen offers several advantages over serum, particularly in endemic regions where blood collection may be challenging due to cultural barriers, pediatric populations, or limited healthcare infrastructure (43). Importantly, urine-based ELISA using Ldocp and Ldairk demonstrated better performance than our previously reported serum-based ELISA using the same antigens. In serum, Ldairk showed sensitivity and specificity of 98.73% and 100%, respectively, while Ldocp demonstrated 96.92% sensitivity and 95% specificity (24, 25). The improved performance observed with urine is likely attributable to its lower protein complexity compared to serum or plasma, facilitating the detection of pathogen-specific antibodies. Additionally, urinary antibodies may exhibit enhanced stability, as they withstand proteolytic degradation during circulation and renal filtration, making them particularly suitable for diagnostic applications (15).

To further advance these findings toward field applicability, we evaluated both antigens in a dipstick format using urine samples from VL and PKDL patients, as well as healthy and disease controls. Both Ldocp-based and Ldairk-based dipsticks showed clear reactivity with VL urine samples, achieving 100% sensitivity. Moreover, no reactivity was observed with negative controls, including endemic and nonendemic healthy individuals and patients with other diseases in the case of Ldairk, whereas minimal nonspecific reactivity was observed with Ldocp among a small number of disease control samples. However, reduced sensitivity was observed with PKDL samples, with Ldocp and Ldairk detecting 50% and 83.3% of cases, respectively. Collectively, these results suggest that Ldairk exhibits higher affinity and diagnostic robustness than Ldocp, particularly in field-based diagnostics. Comparative evaluation with a commercially available rK39 ICT demonstrated that the reference test achieved 100% sensitivity for VL and 83% sensitivity for PKDL when urine samples were used; however, it showed reduced overall specificity of 83% among negative control groups. Limited studies have reported rK39 reactivity in PKDL urine samples, although results remain inconsistent due to small cohort sizes. Notably, Goswami et al. reported rK39 positivity in six urine samples from PKDL patients (44), supporting the feasibility of urine-based antibody detection while highlighting the need for more robust antigens.

In endemic regions, anti-*Leishmania* antibodies may persist for years following clinical cure, potentially due to repeated exposure or incomplete parasite clearance (40). This persistence represents a major limitation of conventional antibody-based diagnostics, as it complicates discrimination between active and past infection. In our longitudinal analysis, Ldairk-specific antibody reactivity in urine declined markedly to approximately 20% by 180 days post-treatment, whereas Ldocp retained reactivity in approximately 80% of samples. Persistent reactivity may signal residual infection or increased relapse risk, warranting continued monitoring. These findings were supported by dipstick assays and contrast favorably with rK39, which retained approximately 80% reactivity six months after treatment, limiting its prognostic utility. Measuring antigen-specific IgG1 responses against Ldocp and Ldairk may further enhance treatment monitoring, as recently reported that IgG1 responses to rK39 decline rapidly after successful treatment and improve outcome monitoring in VL (45). However, longitudinal studies involving larger patient cohorts are required to fully establish the prognostic value of these antigens for treatment monitoring and relapse prediction.

Despite the encouraging results, this study has limitations. The cohort consisted of clinically confirmed VL and PKDL cases, precluding evaluation in suspected or asymptomatic individuals. In addition, samples from patients with related comorbidities, including HIV co-infection, were not included, limiting assessment of assay performance in immunocompromised populations. In addition, the relatively small sample size, particularly for follow-up analyses, may limit the assessment of prognostic performance. Establishing an appropriate cut-off value for ELISAs is often difficult, as it involves balancing sensitivity and specificity, especially when absorbance readings are near the decision threshold. Continued investigation in larger and more diverse cohorts is therefore essential to further refine the Ldocp-based and Ldairk-based rapid diagnostic platform and enhance its suitability for rapid, field-deployable VL diagnostics. Future multicentric validation studies with both antigens across diverse endemic regions, including Africa, are essential to establish diagnostic accuracy, reproducibility, and cross-reactivity with other neglected tropical diseases. Given the epidemiological importance of PKDL as a parasite reservoir that often escapes surveillance (22), further focused evaluation in this group is especially warranted.

In conclusion, our findings demonstrate that recombinant Ldocp and, in particular, Ldairk are promising antigens for noninvasive urine-based diagnosis and treatment monitoring of VL. Refinement and large-scale validation of these diagnostics could significantly enhance VL control by enabling safer, field-deployable, and regionally robust diagnostic tools.

## Data Availability

All the information provided in the research is contained within the article. For additional information, please contact the corresponding author.
